# Nitrite Attenuates the In Vitro Inflammatory Response of Immune Cells to the SARS-CoV-2 S Protein without Interfering in the Antioxidant Enzyme Activation

**DOI:** 10.3390/ijms25053001

**Published:** 2024-03-05

**Authors:** Miguel D. Ferrer, Clara Reynés, Laura Jiménez, Gianluca Malagraba, Margalida Monserrat-Mesquida, Cristina Bouzas, Antoni Sureda, Josep A. Tur, Antoni Pons

**Affiliations:** 1 Research Group on Community Nutrition and Oxidative Stress, University of Balearic Islands-IUNICS, 07122 Palma, Spaingianluca.malagraba@uib.es (G.M.); margalida.monserrat@uib.es (M.M.-M.); cristina.bouzas@uib.es (C.B.); antoni.sureda@uib.es (A.S.); pep.tur@uib.es (J.A.T.); 2 Health Research Institute of Balearic Islands (IdISBa), 07120 Palma, Spain; 3 Centro de Investigación Biomédica en Red Fisiopatología de la Obesidad y la Nutrición (CIBEROBN), Institute of Health Carlos III, 28029 Madrid, Spain

**Keywords:** antioxidants, COVID-19, inflammation, nitric oxide, nitrite, SARS-CoV-2

## Abstract

SARS-CoV-2 induces a hyperinflammatory reaction due to the excessive release of cytokines during the immune response. The bacterial endotoxin lipopolysaccharide (LPS) contributes to the low-grade inflammation associated with the metabolic syndrome, enhancing the hyperinflammatory reaction induced by the SARS-CoV-2 infection. The intake of sodium nitrate, a precursor of nitrite and nitric oxide, influences the antioxidant and pro-inflammatory gene expression profile after immune stimulation with LPS in peripheral blood mononuclear cells from metabolic syndrome patients. We aimed to assess the inflammatory and antioxidant responses of immune cells from metabolic syndrome patients to exposure to the SARS-CoV-2 spike protein (S protein) together with LPS and the effect of nitrite in these responses. Whole blood samples obtained from six metabolic syndrome patients were cultured for 16 h at 37 °C with four different media: control medium, control medium plus LPS (100 ng/mL), control medium plus LPS (100 ng/mL) plus S protein (10 ng/mL), and control medium plus LPS (100 ng/mL) plus S protein (10 ng/mL) plus nitrite (5 µM). Immune stimulation with the LPS/S protein enhanced nitrate biosynthesis from nitrite oxidation and probably from additional organic precursors. In vitro incubations with the LPS/S protein enhanced the expression and/or release of pro-inflammatory TNFα, IL-6, IL-1β, and TLR4, as well as the expression of the anti-inflammatory IL-1ra and IL-10 and antioxidant enzymes. Nitrite attenuated the pro- and anti-inflammatory response induced by the S protein without interfering with the activation of TLR4 and antioxidant enzyme expression, raising the possibility that nitrite could have potential as a coadjutant in the treatment of COVID-19.

## 1. Introduction

Metabolic syndrome (MS) is recognized as a predisposing factor for worse clinical outcomes and death in the coronavirus disease 2019 (COVID-19). A strong association between MS pathologies and the severity of severe acute respiratory syndrome coronavirus 2 (SARS-CoV-2) infection has been reported [1]. SARS-CoV-2 immunopathology can be characterized by an acute hyperinflammatory state that mediates in the complications of the COVID-19, and that may be caused by the excessive release of cytokines, leading to lymphopenia and a disrupted immune response [2]. MS is associated with chronic low-grade inflammation and immune dysregulation, which could exacerbate the hyperinflammatory response in severe COVID-19 cases [2,3]. The translocation of bacterial products, such as the endotoxin lipopolysaccharide (LPS), from the gut into circulation contributes to the low-grade inflammation observed in MS [4,5]. Pre-existing and inducible endotoxemia could also contribute to the severity of COVID- 19 outcomes [6]. In this context, the S protein of SARS-CoV-2 can directly interact in vitro with LPS, thus enhancing nuclear factor kappa B (NF-κB) activation and the cytokine response [7]. The severity of COVID-19 can be related to the level of pro-inflammatory cytokines and subsets of immune cells, among other factors [8]. The early anti-inflammatory treatments to inactivate cytokine functions (mainly anti-interleukin-1 and anti-interleukin-6 compounds) contribute to the improvement of the prognosis of COVID-19 [9,10]. Therefore, reducing the capability of immune cells to produce pro-inflammatory cytokines could contribute to the improvement of COVID-19 in patients presenting hyperinflammation [11].

Viral infection results in the activation of several pattern-recognition receptors (PRRs) [12,13] and toll-like receptors (TLRs), leading to the activation of NF-κB, a transcription factor that promotes the expression of pro-inflammatory cytokines such as interleukin 6 (IL-6) and interleukin 1β (IL-1β) [14,15,16]. The recognition of viral RNAs or the S protein by these PRRs and TLRs can stimulate the production of pro-inflammatory cytokines [12,13]. Thus, the S protein can contribute to the exaggerated local and systemic inflammatory responses and to the pathology observed in severe COVID-19 [17]. Additionally, the interaction between LPS and the S protein can result in the formation of a complex that can trigger NF-κB activation, leading to a subsequent cytokine response in monocytic cells in vitro [7].

There is a growing body of evidence suggesting that dietary supplementation with nitrate may serve as a strategy to alleviate symptoms of MS, enhance vascular function, and mitigate mild inflammatory and oxidative responses [18,19,20,21,22,23]. The intake of sodium nitrate impacts the gene expression profile related to the antioxidant and pro-inflammatory responses following immune stimulation with LPS in peripheral blood mononuclear cells (PBMCs) from individuals with MS [24]. This inflammatory reaction following LPS stimulation indicates a certain degree of tolerance to such stimulation, potentially operating at the level of TLRs [24]. Dietary nitrate intake additionally inhibits the pro-inflammatory effects of acute exercise observed in MS patients, including the elevation of plasma concentrations of tumor necrosis factor α (TNFα), prostaglandin E1 (PGE1), prostaglandin E2 (PGE2), intercellular adhesion molecule 1 (ICAM1), and 16-hydroxy-palmitate (16-HPAL) [21]. It has been pointed out that nitrate intake might attenuate cyclooxygenase and cytochrome P450 activities [21], thus influencing the synthesis of pro-inflammatory oxylipins. In this study, we hypothesized that the exposure of immune cells from MS patients to the SARS-CoV-2 S protein and LPS would trigger an inflammatory response characterized by elevated cytokine levels, and that this response could be regulated by the presence of nitrite. Therefore, the aim of this study was to assess the inflammatory and antioxidant responses of immune cells from MS patients to exposure to the SARS-CoV-2 S protein and LPS, and to investigate the effect of nitrite on these responses.

## 2. Results

### 2.1. Subject Characterization

Blood samples were obtained from six MS patients. The anthropometric data from these patients are presented in Table 1. All the patients were aged over 62 years. Male participants presented a BMI over 30, indicating obesity, while the only female participant presented a BMI corresponding to overweight/pre-obesity according to the World Health Organization (WHO) [25]. Furthermore, two out of the six patients presented arterial hypertension (systolic arterial pressure ≥130 mm Hg and diastolic arterial pressure ≥85 mm Hg).

The blood biochemistry results from these patients are shown in Table 2. One of the patients exhibited hypercholesterolemia and hypertriglyceridemia. Another patient exhibited hypertriglyceridemia without hypercholesterolemia. All the patients exhibited HDL values lower than the reference value for the population (60 mg/dL). One of the patients exhibited an Hba1c value indicative of pre-diabetes, while two of the patients exhibited Hba1c values indicative of diabetes. All participants were diagnosed with MS.

### 2.2. Effects of the Incubation on Nitrite and Nitrate Levels

**Figure 1:**
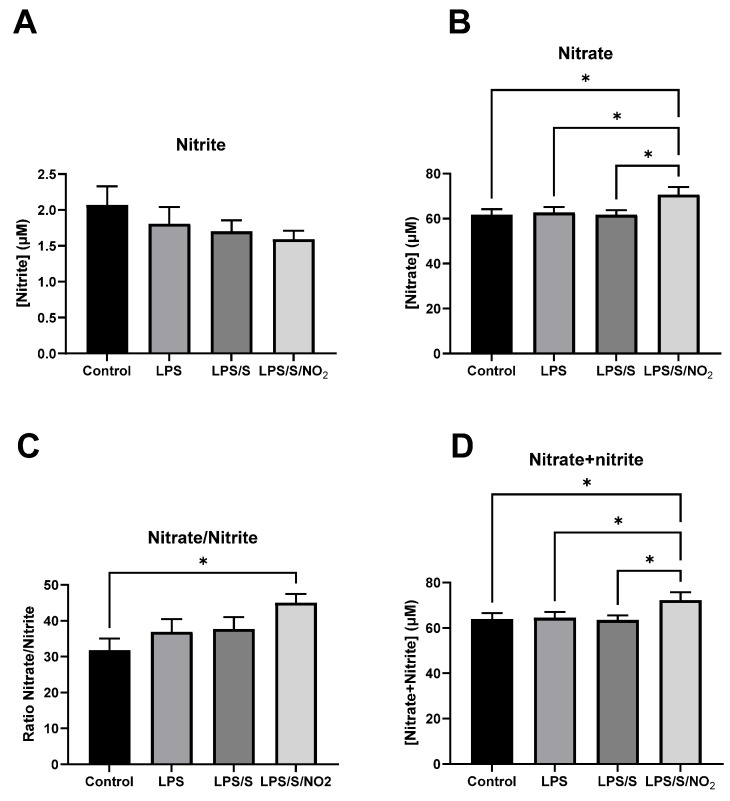
Nitrite and nitrate concentrations in the extracellular medium after incubation of blood cells with LPS (100 ng/mL), LPS (100 ng/mL)/S (10 ng/mL), and LPS (100 ng/mL)/S (10 ng/mL)/NO_2_ (5 µM). (**A**) Nitrite concentration. (**B**) Nitrate concentration. (**C**) Ratio nitrate/nitrite. (**D**) Nitrate plus nitrite concentration. Results represent mean ± SEM. Statistical analysis: ANOVA for repeated measures and two-stage linear step-up procedure of the Benjamini, Krieger, and Yekutieli test to identify the differences between groups. (*) represents significant differences, *q* < 0.05.

The blood samples from each patient were incubated under control conditions or in the presence of LPS (100 ng/mL), LPS (100 ng/mL) with the S protein (10 ng/mL), or LPS (100 ng/mL) with the S protein (10 ng/mL) and NO_2_ (5 µM). After the in vitro incubation of whole blood, nitrite and nitrate concentrations were determined in the extracellular medium (Figure 1). There were no significant differences in nitrite concentration among treatments, indicating that the initially added nitrite at a concentration of 5 µM was depleted after the 16 h incubation period. Consequently, consistent nitrite levels were maintained across all treatments by the end of the incubation period. Regarding nitrate concentration, only the treatment with LPS/S/NO_2_ resulted in an increase in nitrate compared to the control group and the other treatments. The significantly higher nitrate/nitrite ratio in the LPS/S/NO_2_ treatment compared to the other treatments suggests a higher rate of nitrite oxidation to nitrate under these conditions. Total nitrate plus nitrite concentration in the culture media increased significantly following treatment with LPS/S/NO_2_ compared to their levels in the media of other cultures.

### 2.3. Effects of the Incubation on the Expression of Pro-inflammatory Markers

**Figure 2:**
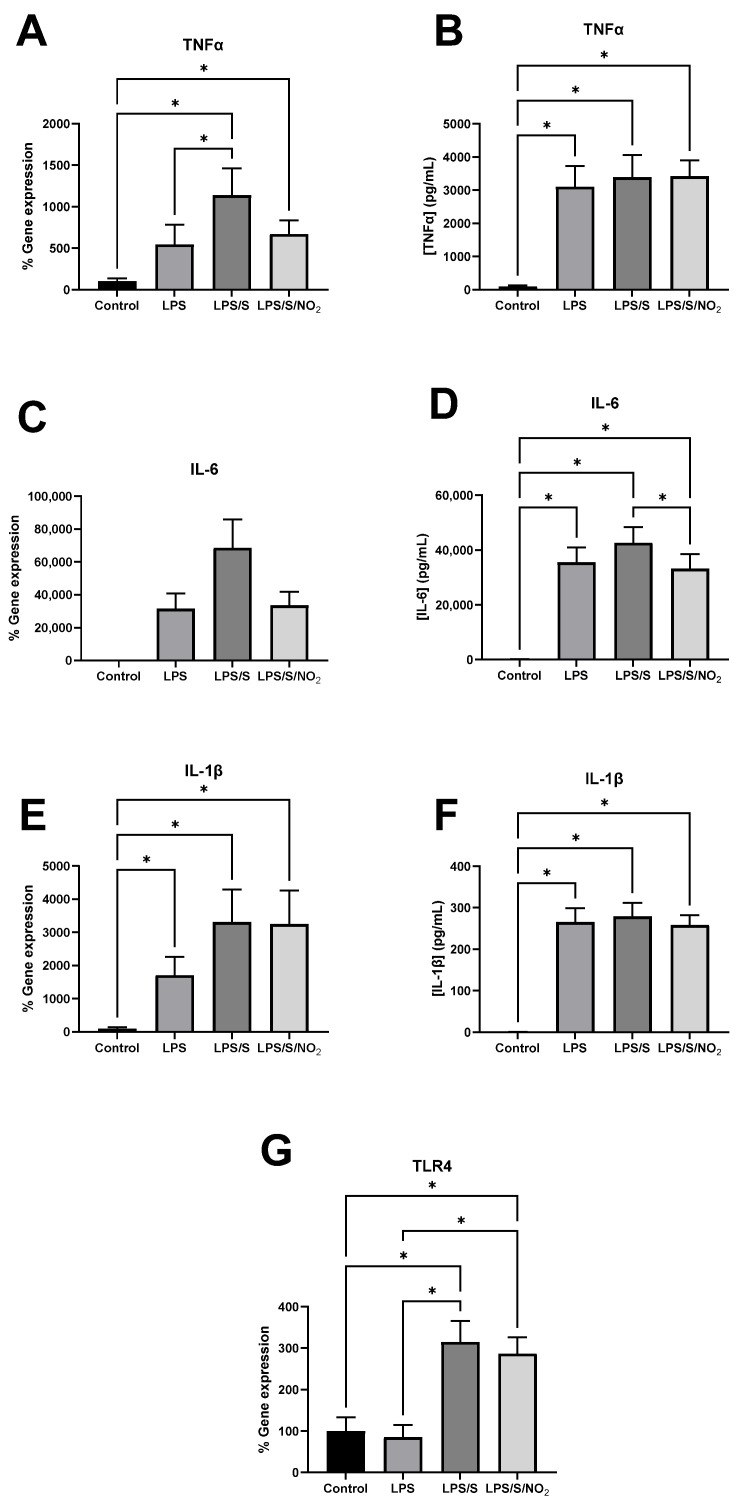
Effect of the incubation of blood cells with LPS (100 ng/mL), LPS (100 ng/mL)/S (10 ng/mL), and LPS (100 ng/mL)/S (10 ng/mL)/NO_2_ (5 µM) on inflammatory marker gene expression in immune cells and concentration in the extracellular medium. (**A**) TNFα gene expression. (**B**) TNFα concentration in the extracellular medium. (**C**) IL-6 gene expression. (**D**) IL-6 concentration in the extracellular medium. (**E**) IL-1β gene expression. (**F**) IL-1β concentration in the extracellular medium. (**G**) TLR4 gene expression. Basal mRNA levels of control samples were arbitrarily set as 100%. The expression of the target genes was normalized with respect to ribosomal 18S. Results represent mean ± SEM. Statistical analysis: ANOVA for repeated measures and when some values where missing data were analyzed by a mixed model. A two-stage linear step-up procedure of the Benjamini, Krieger, and Yekutieli test was performed to identify the differences between groups. (*) represents significant differences, *q* < 0.05.

The gene expressions of inflammatory markers and their corresponding protein concentrations in the culture medium are shown in Figure 2. Incubation with LPS alone did not lead to a significant increase in TNFα gene expression. However, the addition of the S protein to the LPS incubation induced a significant increase compared to both the control conditions and the LPS incubation alone. The addition of NO_2_ to the incubation with LPS and the S protein showed a tendency (*q* = 0.055) to decrease TNFα gene expression, approaching levels observed in the incubation with LPS alone. Regarding IL-6, a significant effect of the incubations was detected. However, only a trend towards increased gene expression following incubation with LPS and LPS with the S protein was obtained after adjusting for multiple testing (*q* = 0.08). A trend to lower IL-6 expression was also observed when NO_2_ was added to the LPS and S protein mixture. All treatments induced a significant increase in IL-1β expression compared to the control condition, with no statistical differences observed among the three treatments. The changes observed in the amount of these cytokines that were secreted into the medium were similar to those observed in the gene expression. All treatments resulted in a similar increase in TNFα and IL1β concentration, with no statistical differences observed between treatments. In this regard, no effects of NO_2_ were observed in the protein levels of TNFα. Regarding IL-6, its concentration was higher than in the control conditions in all treatments. However, the addition of NO_2_ led to a significant decrease in IL-6 concentration compared to treatment with LPS and the S protein. TLR4 expression significantly increased after the incubation with LPS/S but not after the incubation with LPS alone, suggesting that the S protein or the complex LPS-S protein could induce the TLR4 expression. The addition of nitrite did not influence the enhanced TLR4 expression induced by LPS and the S protein.

### 2.4. Effect of the Incubations on the Expression of Anti-Inflammatory and Antioxidant Markers

**Figure 3:**
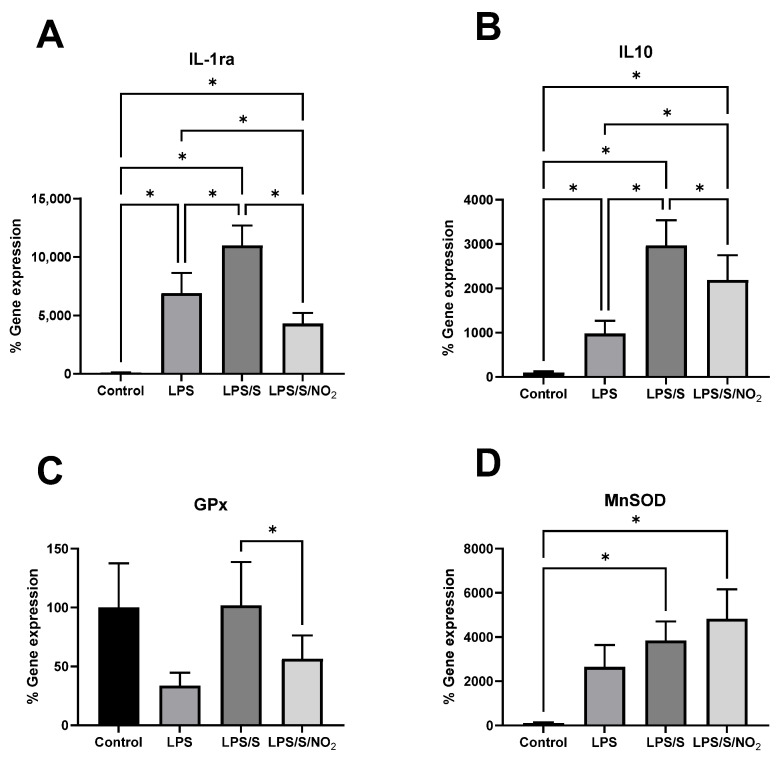
Effect of the incubation of blood cells with LPS (100 ng/mL), LPS (100 ng/mL)/S (10 ng/mL), and LPS (100 ng/mL)/S (10 ng/mL)/NO_2_ (5 µM) on gene expression of anti-inflammatory and antioxidant markers in immune cells. (**A**) IL-1ra gene expression. (**B**) IL-10 gene expression. (**C**) GPx gene expression. (**D**) MnSOD gene expression. Basal mRNA levels of control samples set as 100%. The expression of the target genes was normalized with respect to ribosomal 18S. Results represent mean ± SEM. Statistical analysis: ANOVA for repeated measures and when some values where missing data were analyzed by a mixed model. A two-stage linear step-up procedure of the Benjamini, Krieger, and Yekutieli test was performed to identify the differences between groups. (*) represents significant differences, *q* < 0.05.

The gene expression of anti-inflammatory and antioxidant markers is shown in Figure 3. Incubation of the cells in the presence of LPS enhanced the expression of IL-1ra. Furthermore, the combination of LPS and the S protein led to a higher and significant increase in IL-1ra gene expression. In this case, the addition of NO_2_ significantly decreased IL-1ra expression to levels slightly below those observed after incubation with LPS alone. IL-10 showed a similar expression profile to IL-1ra, with all treatments leading to an increase in IL-10 expression. The addition of the S protein led to higher gene expression compared to incubation with LPS alone, and the addition of NO_2_ decreased this expression. However, the expression of IL-10 after the addition of NO_2_ remained significantly higher than that observed with LPS alone. Regarding the antioxidant enzymes, no effects of any treatment were observed in the expression of glutathione peroxidase (GPx). However, MnSOD expression was significantly upregulated by the combination of LPS and the S protein. The additional presence of NO_2_ under these conditions did not alter this overexpression.

A correlation analysis was performed between the expressions of all analyzed genes (Table 3). The majority of these genes significantly correlated with each other, indicating that the gene activation of pro- and anti-inflammatory proteins and antioxidant enzymes occurs in a coordinated manner. IL-6 expression correlated with the expression of the pro-inflammatory IL-1β and TNFα, as well as the anti-inflammatory IL-10 and IL-1ra, and the antioxidant GPx and MnSOD genes. However, it did not exhibit a correlation with the expression of TLR4. The pro-inflammatory IL-1β expression correlated with the expression of the same pro-inflammatory, anti-inflammatory, and antioxidant genes as IL-6. The expression of TLR4 correlated with the expression of the pro-inflammatory TNFα, the anti-inflammatory IL-10 and IL-1ra, and the antioxidant MnSOD genes. TNFα expression correlated with the expression of all pro-inflammatory, anti-inflammatory, and antioxidant genes, except for IL-1ra. The expression of the anti-inflammatory IL-1ra correlated with all genes, except for TNFα and GPx. MnSOD and IL-10 expression correlated with the expression of all pro-inflammatory, anti-inflammatory, and antioxidant genes. The expression of GPx did not correlate with the expression of TLR4 or IL-1ra genes.

## 3. Discussion

MS typically presents with a chronic inflammation status [26]. This chronic inflammation is characterized by high levels of inflammatory mediators in plasma, such as TNFα, IL-6, and IL-1β [27]. In addition to elevated cytokine levels in circulation, inflammation is commonly accompanied by an increase in the production and release of ROS, prompting the activation of antioxidant enzymes like GPx or MnSOD [28]. Both the pro-inflammatory and oxidative stress status of MS patients may contribute toward COVID-19-mediated host immune dysregulation [29,30]. In this study, we show the impact of an in vitro incubation of blood cells with LPS and the S protein of SARS-CoV-2 on the expression levels and/or release of cytokines and antioxidant enzymes. Likewise, an attempt is made to reduce the response of cytokines and antioxidant enzymes by adding nitrite, which acts as a nitric oxide donor, into the incubation. The study was conducted using blood samples obtained from six individuals diagnosed with MS. This factor should be taken into consideration when interpreting the results.

The levels of nitrite in the medium were not significantly affected after the 16 h of incubation with either LPS alone or LPS with the S protein, nor with the addition of 5 µM nitrite. In all cases, the nitrite concentration in the medium was between 1.5 and 2.0 µM, even after the addition of 5 µM nitrite at the beginning of the incubation. This could point to the partial transformation of nitrite during the incubation of blood cells, especially after immune stimulation with LPS/S in the presence of added nitrite. The estimated half-life of nitrite in the circulation ranges between 15 and 45 min [31,32]. Circulating nitrite incorporates into blood cells or other tissues, where it can be oxidized to nitrate or it can be reduced to nitric oxide [33]. In our study, the nitrite added to the blood cell incubations for 16 h was either oxidized to nitrate or initially reduced to nitric oxide by immune cells, subsequently transforming it into nitrate. This resulted in an increased concentration of nitrate in the extracellular media of the LPS/S/NO_2_ incubation. Immune activation with LPS/S could change the pattern of nitrite transformation to be more oxidative, thus driving the major synthesis of nitrate from nitrite and nitric oxide. However, determining the extent of LPS/S contribution to the observed rise in nitrate levels is challenging without a nitrite-only incubation control. The increase in nitrate in the incubations with LPS/S/NO_2_ cannot be exclusively attributed to the transformation of the added nitrite, which evidences additional sources of nitrate formation. This additional extracellular nitrate may possibly come from nitrate efflux from erythrocytes, from S-nitroso proteins and amino acids, and from the nitric oxide synthase pathway in immune cells. This is consistent with observations that COVID-19 patients experience significant redox imbalance [34,35,36] and elevated nitrate plasma levels [37,38] during infection. This suggests that immune stimulation with LPS/S/NO_2_ enhances nitrate biosynthesis from nitric oxide and nitrite oxidation and, probably, from organic precursors such as S-nitroso thiols and L-arginine through the nitric oxide synthase pathway. In fact, it has been evidenced that immune activation of TLR4 results in marked induction of nitric oxide synthase and nitrite production in microglial cells [39,40] and macrophages [41] in culture.

The cytokine storm, characterized by an excessive release of proinflammatory cytokines, is a determining factor in the pathogenesis of COVID-19 [42]. Several studies have reported an increase in the systemic levels of pro-inflammatory mediators such as TNF-α, along with components of the interleukin family, among patients with COVID-19 requiring intensive care unit (ICU) admission [43,44]. Once the S protein on the virus envelope is recognized by the immune system, both innate and adaptive immunity are activated, leading to the generation of large amounts of pro-inflammatory cytokines and chemokines [17]. In certain patients, this activation becomes so massive that it triggers a cytokine storm, leading to a thrombotic tendency, multi-organ failure, and ultimately, death [42]. MS patients are typically in a state of chronic inflammation, which could accelerate this cytokine storm and lead to a worse prognosis for the disease [30]. In this study, we chose to investigate the effect of the S protein in combination with LPS due to prior evidence indicating that the S protein can bind to LPS [7]. This interaction may play a role in the systemic inflammatory response observed in severe cases of the disease. This decision may represent a limitation of the study, as we do not provide data on the effects of the S protein alone. LPS induces an inflammatory response in PBMCs, increasing the expression and the production rate of TNFα, IL-6, and IL-8 [45]. This is in accordance with the current observations wherein the blood cells obtained from MS patients, when incubated with LPS, activate the expression and release of TNFα and IL-6. The addition of the S protein to the incubation with LPS accentuated the pro-inflammatory response characterized by the increased gene expression of IL-6, IL-1β, and TNFα. However, it must be noted that the release of all these cytokines activated by LPS was not further enhanced by the presence of the S protein. This different behavior between the gene expression and the protein secretion might be explained by the time course of the process. The 16 h of study might not be enough to see differences in the secretion of these cytokines between the LPS and the LPS/S groups. Another possible explanation could be that the protein synthesis and secretion were already saturated following stimulation with LPS, and the additional stimulation with the S protein did not result in increased protein synthesis despite the enhanced gene expression. The concentration of LPS used in this study was higher than the highest concentration used in the study by Petruk et al. [7], which could potentially explain the differences in the observed effects. In this instance, the pro-inflammatory response following stimulation with a high concentration of LPS may already be saturated, meaning that the additional presence of the S protein would not lead to an increase in this response.

The activation of immune cells, such as monocytes and macrophages, by LPS is mediated through its binding to TLR4, initiating the TLR4/NF-κB signaling pathway. This cascade response leads to the activation of inflammatory mediators such as TNF-α and IL-6 [46,47], as well as inducible nitric oxide synthase, consequently increasing nitric oxide production [41,48,49]. Interestingly, TLR4 expression is not affected by LPS alone, confirming that the effects of LPS are independent of the activation of the gene expression of the receptor. However, the combination of LPS and the S protein enhanced the expression of TLR4. This overexpression of TLR4 was accompanied by the increased expression of TNF-α. These results suggest an effect of the S protein on the genetic activation of TLR4, rendering immune cells more sensitive to respond to pro-inflammatory stimuli such as LPS.

The gene expression of IL-10 and IL-1ra, interleukins that participate in the anti-inflammatory response, exhibited a similar pattern of response to the incubations with LPS and LPS/S. Stimulation with LPS induced the expression of both IL-10 and IL-1ra, and the addition of the S protein to the medium further enhanced the IL-10 response. The response observed at the gene expression level in these anti-inflammatory cytokines is similar to that observed in the pro-inflammatory parameters. This similarity suggests that both LPS and LPS/S induced an anti-inflammatory response concurrently or subsequent to the pro-inflammatory response aimed at resolving inflammation. The expression of the pro-inflammatory, anti-inflammatory, and antioxidant genes significantly correlated among them, indicating that the activation of genes encoding pro- and anti-inflammatory proteins and antioxidant enzymes occurs in a coordinated manner [50]. Indeed, the NF𝛋B signaling pathway regulates the expression of a wide range of pro- and anti-inflammatory cytokine genes, as well as antioxidant genes [51,52]. The severity of inflammation could be determined by the balance between pro- and anti-inflammatory cytokines, given that the antagonism between opposing cytokines plays a relevant role in other conditions, such as rheumatoid arthritis [53]. However, critical and severe COVID-19 patients present higher IL-6 and IL-10 plasma levels compared to moderate cases of the disease [54]. This is consistent with the effects of immune cell stimulation with LPS/S, which increases the expression of IL-6 and IL-10 in these blood cells [21]. Moreover, it has been noted that IL-6 can stimulate the production of anti-inflammatory components such as IL-1ra and IL-10 by immune cells. Additionally, TNFα can induce the production of IL-6 by immune cells [55]. Therefore, the correlations observed among IL-6, TNFα, IL-1ra, and IL10 reflect these interactions in the production of both pro-inflammatory and anti-inflammatory cytokines by circulating immune cells stimulated with LPS or LPS/S.

We subsequently investigated how the presence of nitrite could modulate the inflammatory response to the exposure of the immune cells to the S protein. Regarding the effect on the expression of pro-inflammatory cytokines, we observed a certain attenuation of the immune response, as evidenced by a significant reduction in the release of IL-6 into the medium following incubation with nitrite. The overexpression of TNFα observed following the addition of the S protein to the medium already containing LPS was mitigated when nitrite was added to the incubation. Previously, we evidenced that dietary nitrate intake prevents the enhancing effects of acute exercise on the plasma concentration of TNFα, while also reducing the capacity of PBMCs and neutrophils to produce oxylipins [21]. The effects of oral nitrate on cytokine production are mediated by the production of nitric oxide in the nitrate/nitrite/nitric oxide pathway. Similarly, the effects of nitrite on cytokine expression could be mediated by its reduction to nitric oxide. Indeed, the reduction in nitrite to nitric oxide by xanthine oxidase attenuates NADH-oxidase activity in LPS-activated macrophages, thereby modulating the inflammatory response [55]. Additionally, the expression of anti-inflammatory interleukins IL-10 and IL-1ra was downregulated following the addition of nitrite. This attenuation of the anti-inflammatory response by nitrite paralleled the attenuation of the proinflammatory response, suggesting a close interplay between the initiation and resolution of the inflammatory processes.

We finally investigated the antioxidant response of immune cells to stimulation with LPS and the S protein. An upregulation of the gene expression of MnSOD was observed upon addition of the S protein to LPS. The additional presence of nitrite in the culture medium did not alter this antioxidant response to the immune stimulation. The increased MnSOD expression may be mediated by the increased production of reactive oxygen species (ROS) in response to the immune activation caused by LPS and the S protein. The mechanisms by which white blood cells produce ROS include catabolic reactions [28] and the activation of myeloperoxidase and NADH-oxidase in neutrophils [56]. It has been previously reported that the presence of ROS can induce MnSOD expression in HL60 cells in vitro [57]. The activation of this antioxidant response induced by LPS/S stimulation in immune cells is not interfered by nitrite, even though this molecule attenuates the inflammatory response induced by LPS and the S protein.

## 4. Materials and Methods

### 4.1. Study Subjects and Sample Preparation

The study was performed with whole blood samples obtained from six MS patients. The inclusion criteria comprised men and women aged 55–75 years old, with a body mass index (BMI) between 27 and 40 kg/m^2^, who met at least three criteria for MS according to the updated harmonized criteria of the International Diabetes Federation and the American Heart Association and National Heart, Lung, and Blood Institute. Exclusion criteria were (a) active cancer or a history of malignant tumors; (b) documented history of previous cardiovascular disease; (c) impossibility to follow a recommended diet or to carry out physical activity; (d) inability or unwillingness to give informed consent. The study protocol was in accordance with the Declaration of Helsinki, and all procedures were approved by the Research Ethics Committee of the Balearic Islands (reference no. 3560/17 PI).

A total of 4 mL of blood was collected from each patient. Thereafter, each blood sample was divided into four 1 mL aliquots and treated in different conditions to obtain four different treatment groups: control, LPS (Merck KGaA, Darmstadt, Germany), LPS with S protein (recombinant super stable trimer, antibodies-online.com, Limerick, PA, USA), and LPS with S protein and NO_2_. To achieve this, 1 mL of blood was diluted 1:4 with human plasma-like medium (Gibco^TM^, Thermo Fisher Scientific, Waltham, MA, USA) alone for the control groups or with the same medium containing either LPS (100 ng/mL); LPS and S protein (100 ng/mL and 10 ng/mL, respectively); or LPS, S protein, and NaNO_2_ (100 ng/mL, 10 ng/mL and 5 µM, respectively) for each treatment group. Following preparation, the dilutions were incubated at 37 °C for 16 h in a shaker. After the incubation period, a blood clot containing erythrocytes was removed, and the samples were centrifuged at 1700× *g*, 4 °C for 15 min to precipitate the white blood cells. The supernatant obtained after centrifugation, containing plasma diluted in the culture medium, was collected and stored at −80 °C. The precipitate containing the leukocyte fraction was resuspended in 1 mL of Tripure (Roche Diagnostics, Mannheim, Germany) to lyse the cells and isolate RNA.

### 4.2. Nitrite and Nitrate Determination

Nitrite and nitrate concentrations in the culture media collected after the incubation were determined by the Griess method. For nitrite determination, 100 µL of culture medium was incubated for 10 min at room temperature in the presence of 0.025% *N*-(1-Naphthyl)-ethylenediamine (NEDD) and 0.5% sulfanilamide. The absorbance of the samples at 540 nm was then measured using a spectrophotometer (Epoch Biotek, Biotek, Winooski, VT, USA). The procedure was repeated after adding 8% vanadium chloride (VCl_3_) to the samples, which converts nitrate to nitrite. Therefore, by adding VCl_3_ to the reaction, the sum of nitrate and nitrite is measured. Finally, the nitrate concentration was calculated by subtracting the nitrite concentration to the nitrate plus nitrite concentration.

### 4.3. RNA Isolation and Quantitative RT-PCR

The mRNA expression of the genes detailed in Table 4 was determined by a real-time polymerase chain reaction (RT-PCR) with human ribosomal 18S as the reference gene. Total RNA was extracted from each sample using the Tripure (Roche Diagnostics, Mannheim, Germany) extraction method according to the manufacturer’s instructions. The concentration of RNA was quantified using spectrophotometry at 260 nm (Epoch Biotek, Biotek, VT, USA). cDNA was synthesized from 1 µg of total RNA using the TaqMan™ Reverse Transcription Reagents Kit (Applied Biosystems, Waltham, MS, USA) by incubating the RNA samples for 60 min at 42 °C and 5 min at 99 °C. To quantify mRNA expression levels, a LightCycler 96 device (Roche Diagnostics, Mannheim, Germany) and 480 SYBR Green I Master Kit (Roche Diagnostics, Mannheim, Germany) were used. The specific primers used and conditions for each gene determination are shown in Table 4. Relative quantification was performed using standard calculations considering 2^(−ΔΔCt)^. Basal mRNA levels of control samples were arbitrarily set as 100%. The expression of target genes was normalized with respect to ribosomal 18S.

### 4.4. Enzyme-Linked Immunosorbent Assay (ELISA)

The concentration of inflammation-related proteins in the media obtained following each incubation treatment was determined using specific ELISA kits for each protein: Diaclone Human TNF-α ELISA Kit for TNF-α, Diaclone Human IL6 ELISA Kit for IL-6, and RayBIO Human IL-1β ELISA kit. ELISAs were carried out according to the manufacturer’s instructions. The samples were properly diluted to fit within the standard curve, and the absorbance at 450 nm was read using an Epoch Bioteck spectrophotometer (Biotek, Winooski, VT, USA).

### 4.5. Statistical Analysis

Statistical analysis of the data was carried out using GraphPad Prism version 9 (GraphPad Software, La Jolla, CA, USA). An ANOVA for repeated measures was performed to detect the effect of the incubations; when some values where missing, data were analyzed by a mixed model. When a significant effect of the incubations was observed, a two-stage linear step-up procedure of the Benjamini, Krieger, and Yekutieli test was performed to identify the differences between groups and to correct for multiple testing. Correlations between different gene expressions were assessed using Pearson’s correlation coefficient. The significance level was set at *p* < 0.05 (or *q* < 0.05 for the Benjamini, Krieger, and Yekutieli test).

## 5. Conclusions

The SARS-CoV-2 S protein, in the presence of LPS, can upregulate the expression of proinflammatory (TNFα, IL-6, IL-1β, TLR4) and anti-inflammatory (IL-1ra, IL-10) cytokines, as well as antioxidant enzymes (MnSOD), in whole blood from MS patients in vitro. The addition of nitrite attenuated the overexpression of TNFα, IL-6, IL-1ra, and IL-10 without interfering with the activation of MnSOD, TLR4, or IL-1β. These effects of nitrite were only confirmed at the level of protein expression for IL-6, which represents a limitation of the study. Other limitations of the study include the reduced sample size and the absence of an incubation with the S protein alone. These findings warrant further investigation of nitrite for the benefit of individuals with MS suffering from COVID-19.

## Figures and Tables

**Table 1:** Anthropometric data from all participants.

Patient	Sex	Age	Weight (kg)	Height (cm)	BMI (kg/cm^2^)	Systolic Blood Pressure (mm Hg)	Diastolic Blood Pressure (mm Hg)
1	Male	67	95.1	176.5	30.53	130	73
2	Female	68	65.8	155.5	27.21	170	93
3	Male	68	96.2	166	34.91	128	68
4	Male	65	88.1	170.5	30.31	122	77
5	Male	68	109.7	174.5	36.03	170	88
6	Male	63	89.7	165.5	32.75	167	100
Reference value						<130	<85

BMI: Body mass index.

**Table 2:** Blood biochemistry data from all participants.

Measurement	Patient 1	Patient 2	Patient 3	Patient 4	Patient 5	Patient 6	Reference Value
Total cholesterol (mg/dL)	170	124	132	151	110	253	<200
HDL (mg/dL)	46	40	47	37	34	38	≥60
LDL (mg/dL)	95	63	71	65	60	181	<100
Triglycerides (mg/dL)	147	106	73	244	133	172	<149
Erythrocytes (10^6^/mm^3^)	4.89	4.47	4.84	4.73	5.65	5.42	4.50–5.80
Hematocrit (%)	45.2	41.1	46.4	45.3	54.6	51	40.0–50.0
Hb (g/dL)	15	13.7	14.8	15.2	18	16.9	12.5–17.2
Hba1c (%)	5.9	6.3	6	6.7	6.9	5.5	3.8–6.2
Leucocytes (10^3^/mm^3^)	7.1	6.75	7.37	10.5	5.56	6.74	4.00–11.00
Neutrophils (10^3^/mm^3^)	4.12	3.87	3.71	6.28	3.04	3.89	1.8–7.5
Lymphocytes (10^3^/mm^3^)	1.89	1.95	2.33	3.3	1.93	2.07	1.0–4.5
Monocytes (10^3^/mm^3^)	0.84	0.59	0.72	0.64	4	0.55	2.5–13.0
Eosinophils (10^3^/mm^3^)	0.23	0.29	0.53	0.25	0.12	0.17	0.5–7
Basophils (10^3^/mm^3^)	0.02	0.05	0.07	0.07	0.07	0.06	0.0–2.0
Platelets (10^3^/mm^3^)	231	231	190	190	196	214	150.0–400.0

HDL: high-density lipoprotein; LDL: low-density lipoprotein; Hb: hemoglobin; Hba1c: glycated hemoglobin.

**Table 3:** Pearson correlation coefficient (r) between gene expression measured by RT-PCR.

Variable	IL-6	IL-1β	IL-10	TLR4	IL-1ra	TNFα	GPx	MnSOD
IL-6	1	0.537 **	0.724 **	0.246	0.742 **	0.732 **	0.485 **	0.357 *
IL-1β		1	0.645 **	0.062	0.547 **	0.497 **	0.475 **	0.644 **
IL-10			1	0.520 **	0.797 **	0.658 **	0.444 **	0.558 **
TLR4				1	0.611 **	0.337 *	0.147	0.388 *
IL-1ra					1	0.557	0.312	0.508 **
TNFα						1	0.572 **	0.543 **
GPx							1	0.523 **
MnSOD								1

Results represent Pearson correlation coefficient (r). (*) *p* < 0.05, (**) *p* < 0.01. GPx: glutathione peroxidase; IL-1β: interleukin 1β; IL-1ra: interleukin 1 receptor antagonist; IL-6: interleukin 6; IL-10: interleukin 10; MnSOD: manganese superoxide dismutase; TLR4: toll-like receptor 4; TNFα: tumor necrosis factor α.

**Table 4:** Primer sequences and cycle conditions used for PCR.

Gene	RV Sequence	FW Sequence	Cycle Conditions
18S rRNA			95 °C 10 s
	5′-GTGTAATCCGTCTCCACAGA	5′-ATGTGAAGTCACTGTGCCAG	60 °C 10 s
			72 °C 15 s
TNFα			95 °C 10 s
	5′-CTGGTTATCTCTCAGCTCCACGCCATT	5′-CCCAGGCAGTCAGATCATCTTCTCGAA	59 °C 10 s
			72 °C 15 s
IL-6			95 °C 10 s
	5′-GTGTAATCCGTCTCCACAGA	5′- ATGTGAAGTCACTGTGCCAG	63 °C 10 s
			72 °C 15 s
IL-1β			95 °C 10 s
	5′-GGCAGACTCAAATTCCAGCT	5′-GGACAGGATATGGAGCAACA	58 °C 10 s
			72 °C 15 s
GPx			95 °C 10 s
	5′-TTCACCTCGCACTTCTCGAA	5′-TTCCCGTGCAACCAGTTTG	63 °C 10 s
			72 °C 15 s
MnSOD			95 °C 10 s
	5′-TGAACGTCACCGAGGAGAAG	5′-CGTGCTCCCACACATCAATC	60 °C 10 s
			72 °C 12 s
TLR4			95 °C 10 s
	5′-TCAGAGGTCCATCAAACATCAC	5′-GGTCACCTTTTCTTGATTCCA	60 °C 10 s
			72 °C 15 s
IL-10			95 °C 10 s
	5′-CCACGGCCTTGCTCTTGTT	5′-AGAACCTGAAGACCCTCAGGC	58 °C 10 s
			72 °C 15 s
IL-1ra			95 °C 10 s
	5′-CGCTCAGGTCAGTGATGTTAA	5’-GAAGATGTGCCTGTCCTGTGT	56 °C 10 s
			72 °C 15 s

FW: forward; GPx: glutathione peroxidase; IL-1β: interleukin 1β; IL-1ra: interleukin 1 receptor antagonist; IL-6: interleukin 6; IL-10: interleukin 10; MnSOD: manganese superoxide dismutase; rRNA: ribosomal RNA; RV: reverse; TLR4: toll-like receptor 4; TNFα: tumor necrosis factor α.

## Data Availability

The raw data supporting the conclusions of this article will be made available by the authors on request.
